# Elevated plasma soluble lectin-like oxidised low-density lipoprotein receptor 1 as an independent prognostic biomarker in sepsis

**DOI:** 10.1186/s12944-025-02462-4

**Published:** 2025-02-13

**Authors:** Patricia Mester, Charlotte Birner, Stephan Schmid, Martina Müller, Vlad Pavel, Christa Buechler

**Affiliations:** Department of Internal Medicine I, Gastroenterology, Hepatology, Endocrinology, Rheumatology, and Infectious Diseases, University Hospital Regensburg, 93053 Regensburg, Germany

**Keywords:** Sepsis, Soluble LOX-1, Survival, C-reactive protein, Aminotransferase, Bilirubin, Prognosis

## Abstract

**Background:**

Soluble lectin-like oxidised low-density lipoprotein receptor 1 (sLOX-1) is overproduced during inflammation, with its expression and release triggered by C-reactive protein (CRP). As CRP levels are typically elevated in sepsis, this study aimed to investigate whether sLOX-1 levels increase in parallel.

**Methods:**

Plasma sLOX-1 levels of 52 patients with systemic inflammatory response syndrome (SIRS), 45 patients with sepsis, 88 patients with septic shock and 37 controls were measured by ELISA. Associations with CRP, underlying diseases, severe acute respiratory syndrome coronavirus type 2 (SARS-CoV-2) and bacterial infections were analysed.

**Results:**

Plasma sLOX-1 levels were similarly elevated in patients with SIRS, sepsis, or septic shock compared to controls. Plasma sLOX-1 levels did not differ between male and female controls or patients. Plasma sLOX-1 levels were comparable in patients infected with SARS-CoV-2, Gram-negative bacteria, or Gram-positive bacteria. No association was observed between sLOX-1 levels and underlying liver cirrhosis or pancreatitis. Notably, plasma sLOX-1 levels correlated positively with leukocyte and basophil counts but showed no correlation with CRP or procalcitonin. Of clinical relevance, positive correlations were also found with aspartate aminotransferase (AST) and bilirubin levels. Among the 41 patients who did not survive, sLOX-1, AST, and bilirubin levels were significantly higher compared to those of survivors.

**Conclusions:**

Plasma levels of sLOX-1 are elevated in patients with SIRS or sepsis and are significantly higher in non-survivors. Of note, they do not correlate with classical inflammatory markers, suggesting that sLOX-1 may function as an independent prognostic biomarker for predicting poor outcomes in patients with SIRS or sepsis.

## Introduction

Systemic Inflammatory Response Syndrome (SIRS) can be triggered by various factors, leading to an uncontrolled inflammatory response [1]. Sepsis and septic shock are diagnosed based on the Sepsis-3 criteria [2].

During sepsis, the proinflammatory response associated with the “cytokine storm” stimulates the production of free radicals [3, 4, 5]. Oxidative stress, driven by reactive oxygen intermediates, causes oxidation of DNA, proteins, and lipids including those lipids carried by low-density lipoprotein (LDL) [5]. The detrimental effects of oxidized-LDL (ox-LDL) have been extensively studied in cardiovascular diseases [6, 7]. However, LDL oxidation also occurs in infectious diseases, with ox-LDL levels rising significantly in the early stages of severe sepsis [8].

Lectin-like oxidised LDL receptor 1 (LOX-1) is a scavenger receptor that binds and promotes internalisation of a variety of structurally unrelated macromolecules such as bacteria, fibronectin and atherogenic lipoproteins such as ox-LDL [9, 10]. Uptake of ox-LDL by macrophages stimulates foam cell formation, which is thought to be a central pathogenic mechanism in atherosclerotic disease [11].

Certain infectious agents, such as Chlamydia pneumoniae, use the LOX-1 receptor for infection and increase the expression of LOX-1 in vascular cells, thereby promoting the internalisation of ox-LDL [12]. The expression of LOX-1 in endothelial cells, macrophages, platelets and smooth muscle cells is induced in inflammation [10], consistent with the observation that chronic inflammation induces the formation of foam cells [13].

Cell surface LOX-1 can be proteolytically cleaved to release its extracellular domain, the soluble LOX-1 (sLOX-1). LOX-1 shedding is activated by inflammatory cytokines such as tumour necrosis factor (TNF) [14]. C-reactive protein (CRP) is an acute-phase protein whose systemic levels are elevated in inflammation [15, 16]. CRP, by increasing levels of reactive oxygen species in macrophages, activates TNF-alpha converting enzyme, a metalloproteinase described to produce the soluble form of TNF, which also generates sLOX-1 [17].

Proprotein convertase subtilisin/kexin type 9 (PCSK9) is another protein that is induced in patients with sepsis [18, 19, 20, 21]. By binding to the LDL receptor, PCSK9 promotes its degradation, preventing systemic LDL uptake and contributing to hypercholesterolaemia [22]. TNF, lipopolysaccharide and ox-LDL increase PCSK9 secretion by endothelial and smooth muscle cells [23]. Interestingly, PCSK9 induced LOX-1 expression in these cells, which may increase the internalization of ox-LDL contributing to oxidative stress and inflammation [23].

PCSK9 levels are elevated in sepsis, but their associations with disease severity and acute organ failure remain inconclusive [19, 21]. Among patients with sepsis or septic shock, those with SARS-CoV-2 infection exhibit higher plasma PCSK9 levels compared to non-infected patients with other causes of severe illness [19]. However, in COVID-19 patients, serum PCSK9 levels were not associated with disease severity [18].

While PCSK9 is predominantly expressed in hepatocytes, LOX-1 expression in these cells is minimal [22, 24]. Serum sLOX-1 levels are elevated in patients with non-alcoholic fatty liver disease (NAFLD) and correlate positively with the NAFLD activity score [25], reflecting an association with inflammation rather than fibrosis stages [25]. This finding highlights the importance of considering underlying liver disease when analysing plasma sLOX-1 levels in patient cohorts. In 2020, an international expert panel proposed redefining NAFLD as metabolic dysfunction-associated fatty liver disease (MAFLD) [26]. As the cited study was published in 2015 [25], it uses the older NAFLD nomenclature, reflecting the diagnostic criteria of that time.

It is worth noting that the increase in C-reactive protein, the most commonly used clinical marker of inflammation, is reduced in patients with liver cirrhosis in response to infection [27, 28]. Several proteins in the circulation are altered in patients with liver cirrhosis, and this is not limited to proteins mainly produced by the liver [29, 30, 31], and this needs to be taken into account when analysing biomarkers in sepsis. To date, sLOX-1 levels have primarily been studied in patients with cardiovascular diseases, such as myocardial infarction and stroke. These studies have highlighted the potential of sLOX-1 as a biomarker for plaque instability [32, 33]. While CRP, PCSK9, ox-LDL and inflammatory cytokines are increased in severe sepsis [8, 18, 19, 34], systemic sLOX-1 levels have, to our knowledge, not been analysed in sepsis.

LOX-1 expression on immature neutrophils in critically ill COVID-19 patients has been shown to correlate positively with clinical severity, inflammatory cytokines, acute respiratory distress syndrome and thrombosis, suggesting a role for LOX-1 in viral infection [35].

This study investigated plasma sLOX-1 levels in patients with SIRS, sepsis, or septic shock, examining associations with CRP and PCSK9 levels, disease severity, SARS-CoV-2 infection, and underlying liver cirrhosis.

## Materials and methods

### Patients and controls

Between August 2018 and February 2024, we collected plasma samples from 185 patients at the University Hospital of Regensburg. These patients were diagnosed with sepsis (45 patients), septic shock (88 patients), or systemic inflammatory response syndrome (SIRS, 52 patients) due to various causes. Patient classification was based on the Sepsis-3 criteria [2] for sepsis and septic shock.

The SIRS criteria are no longer used to define sepsis. However, some patients in our cohort were admitted with suspected sepsis but did not develop sepsis according to the Sepsis-3 definition. These patients fulfilled at least two of the SIRS criteria [2, 36] and were therefore included in the SIRS group. Although the SIRS criteria are no longer part of the sepsis definition, the Robert Koch Institute still recommends using these criteria for the early recognition of infection [37]. As SIRS and sepsis are different manifestations of a complex disease, the patients were accordingly categorized into SIRS, sepsis and septic shock.

Blood samples were taken within 24 h after intensive care unit (ICU) admission. The patients were assigned to the SIRS, sepsis or septic shock group according to their condition upon admission to the ICU. All patients categorized in the SIRS group were admitted with suspected sepsis but ultimately, according to the Sepsis-3 definition, they did not develop it. The other patients categorized in the sepsis and septic shock group fulfilled the Sepsis-3 criteria already at ICU admission and were accordantly classified [2]. None of the SIRS patients included in this study developed sepsis, and none of the sepsis patients progressed to septic shock. Death cases were only among patients admitted already in a septic shock state.

According to the SIRS criteria, patients categorized in the SIRS group fulfilled at least two of the following criteria: temperature > 38 °C or < 36 °C, heart rate > 90/min, respiratory rate > 20/min or Paco2 < 32 mm Hg (4.3 kPa), white blood cell count > 12 000/mm3 or < 4000/mm3 or > 10% immature bands. These patients had a SOFA score below 2. Patients fulfilling the Sepsis-3 definition were classified in the sepsis group, if the SOFA score was ≥ 2, or in the septic shock group, if vasopressors were required to maintain the mean arterial pressure above 65 mmHg and the serum lactate levels were above 2 mmol/L despite adequate fluid resuscitation.

Principally, sepsis is limited to patients suspected of having an infection. However, several studies have shown that in many cases the causative pathogen remains undetected, some authors reporting up to 70% of cases having negative cultures [38, 39, 40, 41]. Conversely, not all patients with infections develop sepsis [42, 43]. Regarding the number of patients with infections in our study, the numbers represent patients with bloodstream infections.

A number of common comorbidities were identified, including neoplasms such as colorectal cancer and adenocarcinoma, which accounted for 14% of cases. In addition, autoimmune diseases, including Hashimoto’s thyroiditis and Sjögren’s syndrome, were present in 8% of patients. Haematological disorders, particularly acute promyelocytic leukaemia and acute lymphoblastic leukaemia, also accounted for 8%. In addition, 7% of patients were found to be immunosuppressed following organ transplantation and 6% had cholangiosepsis.

Among the patients, 29 COVID-19 patients had plasma samples collected between October 2020 and January 2023. Importantly, all COVID-19 patients included in the study were in sepsis or septic shock. Patients with multidrug-resistant infections, viral hepatitis, or human immunodeficiency virus (HIV) infection were excluded from the study.

The Institute of Clinical Chemistry and Laboratory Medicine at the University Hospital of Regensburg provided the laboratory values, while the Institute of Clinical Microbiology and Hygiene conducted microbiological tests.

### Soluble LOX-1 ELISA and plasma cholesterol

Using EDTA as an anticoagulant, we collected blood samples from patients 12 to 24 h after admission to the intensive care unit for plasma preparation. The human LOX-1/OLR1 DuoSet ELISA (Biotechne; Wiesbaden, Nordenstadt, Germany) was used for analysis according to the manufacturer’s instructions. Soluble LOX-1 levels were measured in undiluted plasma in duplicate and the mean values were used for calculations. Cholesteryl ester and free cholesterol levels were measured in a sub-cohort of patients [44] and were analysed by flow injection analysis Fourier-transform mass spectrometry on a high-resolution hybrid quadrupole-Orbitrap mass spectrometer as described [44, 45].

Analysis of plasma PCSK9 levels was described before [19].

### Statistical analysis

Patient plasma sLOX-1 levels were not normally distributed, as confirmed by the Kolmogorov-Smirnov and Shapiro-Wilk tests (*p* < 0.001 for both tests). Data are presented using box plots, which display the median, first and third quartiles, and the minimum and maximum sLOX-1 values. Outliers are indicated with asterisks or single circles. Median, minimum, and maximum values are additionally summarised in the tables.

Statistical analyses were conducted using IBM SPSS Statistics 26.0 software (IBM Corp. Released 2019. IBM SPSS Statistics for Windows, Version 26.0. Armonk, NY: IBM Corp). Non-parametric tests, including the Mann-Whitney U test and Kruskal-Wallis test, were applied for group comparisons. The Chi-square test was used for categorical variables, while receiver operating characteristic (ROC) curve analysis and Spearman’s correlation assessed diagnostic performance and relationships between variables, respectively. The Youden´s statistic was used to determine the optimal cut-off values. Statistical significance was defined as *p* < 0.05.

## Results

### Plasma sLOX-1 in patients and controls

The study included 37 controls (19 males and 18 females with a median age of 57 (24–81) years) and 185 patients with SIRS, sepsis or septic shock. The controls were of similar age but included more females (*p* = 0.010) compared to the patients. All controls were of normal weight and good health, and thus laboratory values of the controls were not recorded. The characteristics of the patients are shown in Table 1.

**Table 1:** Characteristics of SIRS/sepsis patients. Superscript numbers are used when data were not available for the whole cohort

Parameters	Patients
Males / Females	137 / 48
Age (years)	60 (19–93)
Body Mass Index (kg/m^2^)	27.0 (15.4–54.5)
SIRS / Sepsis / Septic Shock	52 / 45 / 88
C-reactive protein mg/l	152 (4–697)
Procalcitonin ng/ml	1.48 (0.05–270.00)
Leukocytes n/nl	10.30 (0.06–1586.00)
Neutrophils n/nl	7.90 (0–70.20)
Basophils n/nl	0.04 (0–0.90)
Eosinophils n/nl	0.08 (0–8.80)
Monocytes n/nl	0.73 (0–45.00)
Lymphocytes n/nl	0.94 (0.08 − 28.60)
Immature Granulocytes n/nl	0.15 (0–7.25)
Aspartate aminotransferase U/l	48 (6–8084) ^170^
Alanine aminotransferase U/l	32 (5–1042) ^167^
Albumin g/l	24.3 (6.3–42.0) ^171^
Gamma glutamyltransferase U/l	142 (11–1266) ^153^
Total Bilirubin mg/dl	1.00 (0.10–36.60) ^175^
Direct Bilirubin mg/dl	0.70 (0.10–30.50) ^173^
Indirect Bilirubin mg/dl	0.30 (0.10–7.80) ^157^

Soluble LOX-1 (sLOX-1) levels were comparable between men and women (*p* = 0.498 for controls, *p* = 0.829 for patients). Plasma sLOX-1 levels showed no correlation with age in controls (*r* = -0.291, *p* = 0.080) but were negatively correlated with age in patients (*r* = -0.205, *p* = 0.005). Among patients, plasma sLOX-1 levels did not correlate with body mass index (*r* = -0.056, *p* = 0.455).

**Fig. 1:**
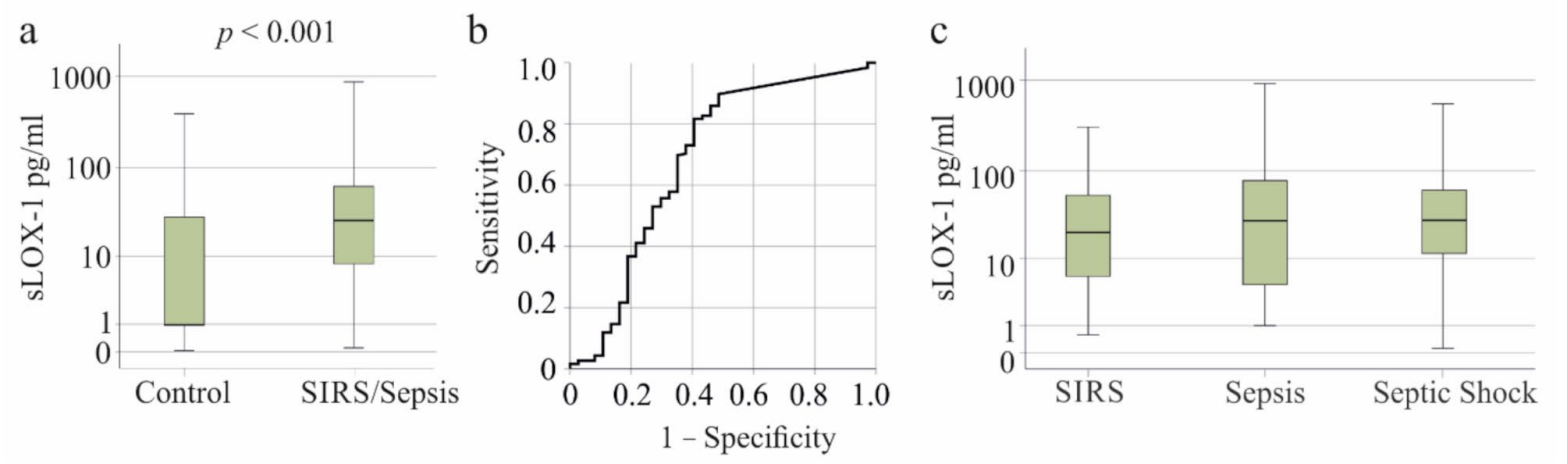
Soluble LOX-1 (sLOX-1) levels in the plasma of patients with SIRS/sepsis and controls. **a** sLOX-1 levels in the plasma of controls (the median of the control group is 1 and is identical to the lower quartile Q1) and patients with SIRS/sepsis. **b** ROC curve for discrimination of controls and patients. **c** sLOX-1 levels in the plasma of patients with SIRS, sepsis and septic shock

In patients with SIRS/sepsis, sLOX-1 levels were significantly elevated at 26.8 (0.1–911.3) pg/ml compared to controls, who had levels of 1.0 (0.05–722.7) pg/ml (*p* < 0.001) (Fig. 1a). The area under the receiver operating characteristic curve (AUROC) for discriminating between controls and patients was 0.685 ± 0.057 (*p* < 0.001) (Fig. 1b). The sensitivity for discriminating between controls and patients at 5.03 pg/ml sLOX-1 was 82% and the specificity was 60%.

Patients with SIRS (*n* = 52), sepsis (*n* = 45), and septic shock (*n* = 88) exhibited similar plasma sLOX-1 levels (*p* = 0.717) (Fig. 1c).

### Patient plasma sLOX-1 in relation to underlying liver cirrhosis, pancreatitis as well as plasma cholesterol levels and measures of liver function

**Fig. 2:**
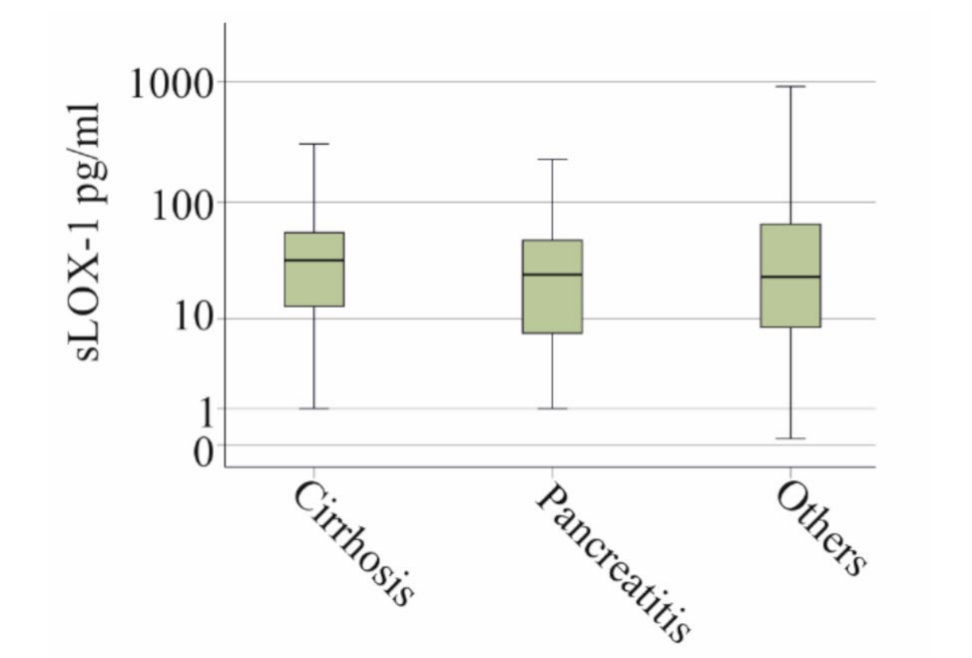
Soluble LOX-1 (sLOX-1) in plasma of patients with liver cirrhosis, necrotizing pancreatitis, and patients with other underlying diseases (others)

Liver cirrhosis (27 patients) and pancreatitis (33 patients) were the most common underlying diseases, affecting more than 10% of the patients. It is worth to note that most of our patients had necrotizing pancreatitis and were in a septic state with organ failure and required vasopressors. Plasma sLOX-1 levels in patients with liver cirrhosis and in patients with necrotizing pancreatitis were comparable to patients with other underlying diseases (*p* = 0.796) (Fig. 2).

Although sLOX-1 levels did not change in liver cirrhosis, plasma sLOX-1 levels correlated positively with aspartate aminotransferase (AST) and with total, direct, and indirect bilirubin, both in the whole cohort and after excluding patients with liver cirrhosis (Table 2).

The liver is the main organ for cholesterol metabolism [46]. Plasma cholesterol and sLOX-1 levels were known for 146 patients (Table S1), 28 of whom had liver cirrhosis. Although total cholesterol levels decrease during severe illness [47, 48], and were 4116 (3330–6526) nmol/ml in plasma of controls and 2339 (712–8529) nmol/ml in the plasma of the patients (*p* < 0.001), plasma cholesterol levels were not associated with sLOX-1 levels (Table 2).

**Table 2:** Spearman correlation coefficients (r) and *p*-values for the correlations between plasma sLOX-1 levels and total plasma cholesterol levels and clinical markers of liver disease

Cholesterol and biomarkers of liver function	All patients		Patients with liver cirrhosis excluded	
	r	*p*-value	r	*p*-value
Total Cholesterol nmol/ml	-0.064	0.441	-0.054	0.559
Aspartate aminotransferase U/l	0.211	0.006	0.218	0.010
Alanine aminotransferase U/l	0.097	0.211	0.124	0.148
Albumin g/l	0.022	0.773	0.042	0.624
Gamma glutamyltransferase U/l	0.056	0.490	0.085	0.344
Total Bilirubin mg/dl	0.267	< 0.001	0.301	< 0.001
Direct Bilirubin mg/dl	0.264	< 0.001	0.285	0.001
Indirect Bilirubin mg/dl	0.263	0.001	0.300	0.001

### Patient plasma sLOX-1 in relation to infections

**Fig. 3:**
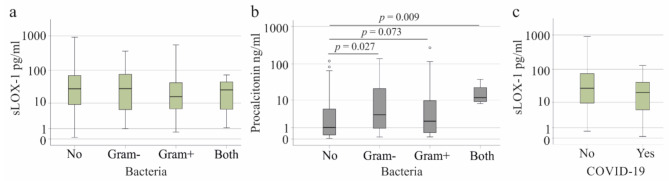
Soluble LOX-1 (sLOX-1) in plasma of patients stratified for bacterial and SARS-CoV-2 infection. **a** sLOX-1 in plasma of patients without, with Gram-negative, Gram-positive or both types of bacteria in the blood. **b** procalcitonin in plasma of patients without, with Gram-negative, Gram-positive or both types of bacteria in the blood. **c** sLOX-1 in plasma of patients with and without COVID-19

Bloodstream infection with Gram-negative bacteria (24 patients), Gram-positive bacteria (35 patients) or both types (4 patients) did not alter plasma sLOX-1 levels (*p* = 0.653) (Fig. 3a). Procalcitonin is a marker for bacterial infections [49] and was higher in plasma of patients with bacterial infections, and this was significant for non-infected compared to Gram-negative infection and infection with both types of bacteria (Fig. 3b). The 29 patients with SARS-CoV-2 infection had similar sLOX-1 levels to those without the virus (*p* = 0.190) (Fig. 3c).

### Correlation of plasma sLOX-1 with leukocyte count, CRP, procalcitonin and PCSK9

C-reactive protein (CRP) has been shown to increase LOX-1 production and release from macrophages [17]. However, CRP levels did not correlate with plasma sLOX-1 levels (Table 2). Although patients with liver cirrhosis typically exhibit low CRP levels [27], excluding these patients did not alter this finding (*r* = -0.072, *p* = 0.374). Positive correlations were observed between plasma sLOX-1 levels and both basophils and immature granulocytes (Table 3). Negative correlations between leukocyte number and sLOX-1 levels were also found (Table 3).

**Table 3:** Spearman correlation coefficients (r) and *p*-values for correlations between plasma sLOX-1 levels and clinical markers of inflammation and PCSK9

Biomarker of inflammation	*r*	*p*-value
Procalcitonin ng/ml	0.119	0.108
C-reactive protein mg/l	-0.071	0.334
Leukocytes n/nl	-0.149	0.043
Neutrophils n/nl	0.130	0.082
Basophils n/nl	0.159	0.033
Eosinophils n/nl	-0.059	0.430
Monocytes n/nl	0.137	0.067
Lymphocytes n/nl	0.116	0.122
Immature Granulocytes n/nl	0.149	0.047
PCSK9 ng/ml	-0.031	0.684

A recent study demonstrated that PCSK9 induces LOX-1 expression [23]. In a previous study conducted by our group, PCSK9 levels were analysed [19] and plasma sLOX-1 and PCSK9 levels were analysed in 175 of these patients (Table S1) This previous cohort was not identical to the current cohort as new plasma samples have been collected in the meantime and plasma collected early in the study has been completed. No correlation was found between sLOX-1 and PCSK9 in the entire cohort (Table 3) and after excluding the 30 patients with liver cirrhosis (*r* = − 0.051, *p* = 0.543). While COVID-19 has been associated with elevated PCSK9 levels [19], sLOX-1 and PCSK9 did not correlate in SIRS/sepsis patients after excluding both, patients with liver cirrhosis and those with COVID-19 (*r* = -0.080, *p* = 0.385).

### Plasma sLOX-1 levels and survival

**Fig. 4:**
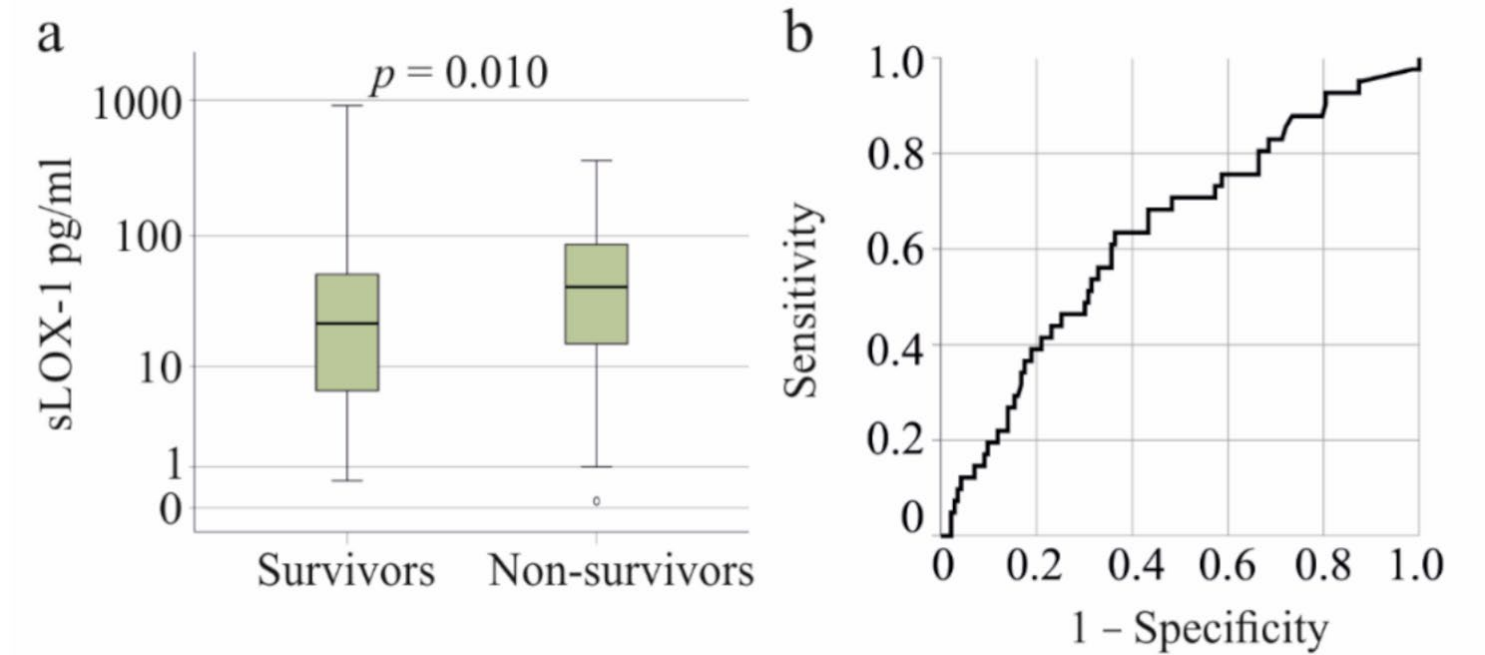
Soluble LOX-1 (sLOX-1) levels in plasma of SIRS/sepsis patients who survived and who died. **a** sLOX-1 in plasma of survivors and non-survivors. **b** ROC curve for discrimination of survivors and non-survivors

In the SIRS/sepsis cohort, higher plasma sLOX-1 levels were observed in the 41 non-survivors compared to survivors (*p* = 0.010, Fig. 4a). Plasma sLOX-1 demonstrated prognostic value for mortality, with an AUROC of 0.633 ± 0.050 (*p* = 0.010) (Fig. 4b). The sensitivity for discriminating between controls and patients at 31.17 pg/ml sLOX-1 was 63% and the specificity was 64%.

In the SIRS/sepsis cohort, non-survivors exhibited significantly higher levels of total bilirubin, direct bilirubin, indirect bilirubin, and AST compared to survivors (Table 4). Total cholesterol levels, however, were comparable between survivors and non-survivors (Table 4).

**Table 4:** Bilirubin, AST and cholesterol levels in plasma of survivors and non-survivors

	Survivors	Non-Survivors	Number of Non-Survivors	*p*-value	AUROC ± Std. dev.	*p*-value AUROC
Total bilirubin mg/dl	0.80 (0.10–36.60)	1.80 (0.20–23.90)	40	0.015	0.664 ± 0.054	0.004
Direct bilirubin mg/dl	0.60 (0.10–30.50)	1.75 (0.20–20.10)	38	0.001	0.685 ± 0.051	0.001
Indirect bilirubin mg/dl	0.30 (0.10–6.70)	0.50 (0.10–7.80)	34	0.042	0.605 ± 0.061	0.066
AST U/l	44 (6–3252)	64 (18–8084)	39	0.017	0.623 ± 0.052	0.031
Cholesterol nmol/ml	2344 (712–8529)	2328 (904–5727)	34	0.363	-	-

## Discussion

This study highlights the potential role of plasma sLOX-1 as a biomarker in SIRS/sepsis, showing elevated levels in patients and an association with mortality. Unlike CRP and procalcitonin, sLOX-1 appears to reflect different aspects of the inflammatory response, warranting further investigation into its clinical utility.

CRP, a marker elevated in inflammation, has been shown to induce LOX-1 shedding from macrophages [17, 50]. Our study demonstrates that plasma sLOX-1 levels in SIRS/sepsis are independent of traditional inflammatory markers like CRP and procalcitonin, contrasting with preclinical evidence suggesting a role for inflammation in LOX-1 shedding [14, 17, 51]. These findings imply that mechanisms beyond inflammation may underlie sLOX-1 elevation in sepsis, warranting further research into its regulation and clinical implications.

In sepsis, ox-LDL levels rise early in the disease course [52], leading to increased LOX-1 expression on the cell surface and subsequent proteolytic shedding of its ectodomain [53]. A decrease in plasma membrane cholesterol levels has been shown to enhance LOX-1 shedding and, by disrupting lipid-rich membrane domains, impairs LOX-1-mediated ox-LDL uptake [54]. Plasma cholesterol levels are low in SIRS/sepsis patients [44], consistent with other studies, and contribute to cellular cholesterol depletion [55]. Consequently, the combined effects of hypocholesterolemia and elevated ox-LDL levels in SIRS/sepsis likely drive the observed increase in soluble LOX-1 (sLOX-1).

Our study suggests a dual role of elevated ox-LDL and hypocholesterolemia in driving sLOX-1 elevation in SIRS/sepsis. Early ox-LDL increases enhance LOX-1 shedding, while low plasma and cellular cholesterol levels contribute to impaired membrane integrity and further promote shedding. These findings suggest a complex interplay between lipid metabolism and LOX-1 regulation in sepsis.

Soluble LOX-1 was similar in patients with and without SARS-CoV-2 and with and without bacterial infections. Rapid and reliable tests have been developed to diagnose SARS-CoV-2 infection [56]. Diagnosis of bacterial bloodstream infections takes several days and the pathogen may not be identified in up to 70% of cases [38, 39, 40, 41]. This is a limitation of studies looking for biomarkers for early detection of bacterial infections. Procalcitonin was found to be such a marker [49] and was elevated in our infected patients compared to non-infected patients, suggesting that the diagnosis of bloodstream infections was mostly correct.

Plasma sLOX-1 levels remain unaffected by liver dysfunction, as evidenced by their similarity in patients with liver cirrhosis and those with other SIRS/sepsis-related conditions. However, the observed correlation between sLOX-1 levels and liver function markers (AST and bilirubin) hints at a potential link, warranting further investigation into the role of liver function in sLOX-1 regulation.

Bilirubin, a byproduct of haemoglobin breakdown, exists in indirect and direct forms, with elevated direct bilirubin levels indicating impaired hepatic clearance [57]. Soluble LOX-1 correlates with both bilirubin forms, but the observational nature of the data prevents definitive conclusions about its association with liver function or haemoglobin metabolism.

Mild and moderate hyperbilirubinaemia is associated with mortality in sepsis [58, 59]. The underlying causes of hyperbilirubinemia in patients with sepsis and poor prognosis are still unresolved [58]. Moderate elevations in AST, ALT, and γ-glutamyl transpeptidase have been associated with increased mortality in patients with infections [60]. However, it remains unclear whether these enzymes originate from the liver, non-hepatic tissues, or both [61].

In our cohort, plasma sLOX-1, AST, and bilirubin levels were elevated in non-surviving SIRS/sepsis patients. Previous studies have demonstrated associations between higher sLOX-1 levels and non-survival in acute coronary syndrome [62, 63, 64], suggesting that sLOX-1 may be linked to survival outcomes in both cardiovascular and infectious diseases. Nonetheless, the AUROC for sLOX-1 in predicting mortality was too low to recommend its use as a standalone predictive marker. AST and bilirubin levels are also not good prognostic markers for survival.

Soluble sLOX-1 levels correlated positively with basophil and immature granulocyte counts. LOX-1 is expressed by neutrophils [65], and LOX-1-expressing immature neutrophils have been linked to disease severity in SARS-CoV-2 infection [35]. Whether this connection explains the correlation between sLOX-1 levels and immature granulocytes requires further investigation, as does the relationship between sLOX-1 and basophil numbers.

LOX-1 and PCSK9 have been shown to positively influence each other’s expression, particularly during inflammatory responses [23]. However, in SIRS/sepsis patients, no correlation was observed between sLOX-1 and PCSK9 levels.

In SIRS/sepsis patients, serum sLOX-1 levels were negatively correlated with age. This contrasts with findings in coronary artery disease patients, where sLOX-1 levels correlated positively with age [66], and in healthy controls, where plasma sLOX-1 levels increased with age in both sexes [67]. Interestingly, in patients with acute coronary syndrome, sLOX-1 levels were unaffected by age [68]. These observations suggest that the relationship between sLOX-1 and age may be influenced by disease-specific factors in severe illness.

This study has several limitations. Plasma levels of ox-LDL and LDL were not analysed. The patients were from southern Germany and the results may not apply to other ethnic groups. Common comorbidities such as type 2 diabetes and atherosclerosis were not documented. It is worth noting that patients with stable cardiovascular disease have normal levels of sLOX-1 in the serum, which are increased in patients with acute coronary syndrome [69]. The increase in sLOX-1 levels in patients with type 2 diabetes is marginal compared to its increase in sepsis [69, 70]. The laboratory values of the controls were not recorded. However, none of our controls were overweight or obese, and all were in good health.

## Conclusions

Elevated plasma sLOX-1 levels in SIRS and sepsis are associated with increased mortality and may thus serve as novel prognostic biomarker in SIRS/sepsis patients. Independent from classical inflammatory markers, sLOX-1 levels provide insights into disease severity and could enhance risk stratification for these patients.

## Data Availability

All data generated or analysed during this study are included in this published article.

## Abbreviations

AST: Aspartate aminotransferase
CRP: C-reactive protein
ox-LDL: Oxidized low-density lipoprotein
PCSK9: Proprotein convertase subtilisin/kexin type 9
sLOX-1: Soluble lectin-like oxidised low-density lipoprotein receptor 1
